# Outcomes of a community-based wellness screening tool administered by mental health professionals and religious leaders in the Ketu South Municipality in Ghana

**DOI:** 10.1192/j.eurpsy.2023.750

**Published:** 2023-07-19

**Authors:** R. P. F. Wolthusen, P. Andrä

**Affiliations:** 1 Psychiatry, Duke University Medical Center, Durham, United States; 2 On The Move e.V., Dresden, Germany; 3 University Children Hospital Zurich, Zurich, Switzerland

## Abstract

**Introduction:**

Ghanaian community members with mental health conditions are usually not identified until their families cannot handle their care at home anymore, for example, due to mistrust in medical institutions. From community-based and global mental health research, we know *why* we should act (for example, early interventions improve the treatment outcomes) and *what* we should do (for example, task-sharing in community settings). *How* any of these activities can be implemented on the community level to decrease the delay of access to evidence-based care remains unclear.

**Objectives:**

The study explored the “how” for a specific identified problem (collaboration between mental health professionals and religious leaders) in the Ketu South Municipality in Ghana; additionally, the study explored the feasibilty and the results of a community-based wellness screening.

**Methods:**

We used a human-centered design approach to tackle this challenge in the Ketu South Municipality in Ghana. We invited 80 mental health professionals, religious leaders, and service users to participate in this exercise. The participants innovated the so-called *Brain Spirit Desk*, which builds collaboration between mental health professionals and religious leaders. The participants also designed a 9-question wellness screening tool, including four validated screening scales in Ghana: PHQ-2, GAD-2, one question about suicidality, and CAGE-AID. The participating religious leaders were trained to use this screening tool and administer it by themselves or allow mental health professionals to administer it in their respective institutions. Referral pathways were established for community members who screened positive on the wellness screening tool.

**Results:**

1065 community members (787 females, 278 males, mean age: 32.42 years) were screened using the wellness screening tool over five months (January - May 2022); 215 of these community members were already connected to mental health clinics in hospitals. 60 community members out of 203 who screened positive on the PHQ-2 were not receiving treatment at the time of screening and were referred for further assessment and treatment. Another 52, 53, and 142 community members were referred for further evaluation and treatment based on their answers to the GAD-2, suicidality, and CAGE-AID screening questions, respectively. Completed referrals across conditions averaged around 55%.

**Conclusions:**

Our activities explored, guided through principles of a human-centered design approach, how the delay in access to evidence-based mental health care in the Ketu South Municipality in Ghana can be decreased through a collaborative effort of mental health professionals and religious leaders. A developed screening tool identified potential cases of mental health conditions. Importantly, religious leaders’ involvement and endorsement built trust in the activities.

**Disclosure of Interest:**

None Declared

